# A Transcriptomic Analysis of Cave, Surface, and Hybrid Isopod Crustaceans of the Species *Asellus aquaticus*

**DOI:** 10.1371/journal.pone.0140484

**Published:** 2015-10-13

**Authors:** Bethany A. Stahl, Joshua B. Gross, Daniel I. Speiser, Todd H. Oakley, Nipam H. Patel, Douglas B. Gould, Meredith E. Protas

**Affiliations:** 1 Department of Biological Sciences, University of Cincinnati, Cincinnati, Ohio, United States of America; 2 Department of Biological Sciences, Florida Atlantic University, Jupiter, FL, 33458, United States of America; 3 Department of Biological Sciences, University of South Carolina, Columbia, SC, United States of America; 4 Department of Ecology, Evolution, and Marine Biology, University of California Santa Barbara, Santa Barbara, CA, United States of America; 5 Department of Molecular and Cell Biology & Department of Integrative Biology, University of California, Berkeley, CA, United States of America; 6 Departments of Ophthalmology and Anatomy, Institute for Human Genetics, UCSF School of Medicine, San Francisco, CA, United States of America; 7 Department of Natural Sciences and Mathematics, Dominican University of California, San Rafael, CA, United States of America; CNRS, UMR 9197, FRANCE

## Abstract

Cave animals, compared to surface-dwelling relatives, tend to have reduced eyes and pigment, longer appendages, and enhanced mechanosensory structures. Pressing questions include how certain cave-related traits are gained and lost, and if they originate through the same or different genetic programs in independent lineages. An excellent system for exploring these questions is the isopod, *Asellus aquaticus*. This species includes multiple cave and surface populations that have numerous morphological differences between them. A key feature is that hybrids between cave and surface individuals are viable, which enables genetic crosses and linkage analyses. Here, we advance this system by analyzing single animal transcriptomes of *Asellus aquaticus*. We use high throughput sequencing of non-normalized cDNA derived from the head of a surface-dwelling male, the head of a cave-dwelling male, the head of a hybrid male (produced by crossing a surface individual with a cave individual), and a pooled sample of surface embryos and hatchlings. Assembling reads from surface and cave head RNA pools yielded an integrated transcriptome comprised of 23,984 contigs. Using this integrated assembly as a reference transcriptome, we aligned reads from surface-, cave- and hybrid- head tissue and pooled surface embryos and hatchlings. Our approach identified 742 SNPs and placed four new candidate genes to an existing linkage map for *A*. *aquaticus*. In addition, we examined SNPs for allele-specific expression differences in the hybrid individual. All of these resources will facilitate identification of genes and associated changes responsible for cave adaptation in *A*. *aquaticus* and, in concert with analyses of other species, will inform our understanding of the evolutionary processes accompanying adaptation to the subterranean environment.

## Introduction

Cave animals have long interested evolutionary biologists [1]. With reduced eyes and pigmentation, elongated appendages, enhanced mechanosensory structures, and unique life history traits, they are unusual and interesting animals (reviewed in [2, 3]). Strikingly, many of these cave characteristics are found in multiple different species, including vertebrates and invertebrates [2, 3]. Therefore, cave animals not only provide a system for the study of many unique characteristics, but also allow comparisons of these traits across distantly- and closely-related species. Furthermore, the study of cave animals allows for examination of characters that are reduced, for example eye loss and pigment loss, but also allows for the examination of gains such as longer appendages, longer life spans, larger embryos, and enhanced sensory features such as taste buds in cave vertebrates and longer chemosensory cells in invertebrates [4, 5, 6, 7].

Studying cave animals, especially from a genetic perspective, has been challenging historically because of the difficulties in generating molecular tools and genetic resources in non-model species. However, the advent of next-generation sequencing technology, alongside reduced costs and higher throughput techniques, enable advanced genetic analyses in virtually any species. As a result, transcriptomes are now available for a number of cave-dwelling animals including a cave beetle, a cave remipede crustacean, and three different species of cave fish [8, 9, 10, 11, 12].

The majority of genetic resources, including genetic markers, genomic sequences, and transcriptomic sequences, have been developed for *Astyanax mexicanus*, a characid fish found in Mexico. Surface and cave-dwelling populations interbreed and produce fertile offspring (reviewed in [13]). Molecular resources available for this species include a draft genome, sequenced transcriptomes for tissues from various life stages and populations, and numerous well-populated linkage maps [14, 10, 11, 15]. In addition, many years of study provide much information about the genetic, developmental, and taxonomic underpinnings of this system (Reviewed in [3]). Although *Astyanax* has provided insight into many of the outstanding questions in cave evolution, the degree to which this information can be applied to other cave organisms remains unclear. For instance, to what extent are different genetic pathways and selective pressures responsible for adaptation in other species of cave animals? To address this question, it is necessary to develop resources for additional cave-dwelling species.

Another cave-dwelling species that has the potential to be an excellent genetic model is *Asellus aquaticus*, an isopod crustacean found throughout Europe. Similar to *Astyanax mexicanus*, this species has both cave and surface dwelling populations that can interbreed and produce fertile offspring [16]. The presence of distinct (but able to interbreed) morphotypes renders *A*. *aquaticus* a promising species for comparisons with *Astyanax mexicanus*. Existing resources for *A*. *aquaticus* include a linkage map and hundreds of genetic markers [17, 18]. In addition, multiple loci responsible for eye and pigmentation traits have been mapped [17]. One locus was observed each for the following five pigmentation traits: presence versus absence of pigmentation, red versus orange and brown pigmentation, light versus dark pigmentation, and stellate versus diffuse pigmentation. In addition, we mapped a locus responsible for eye presence versus absence (a qualitative trait) and a different locus responsible for eye size (a quantitative trait).

However, there is no genome sequence or catalogue of expressed sequence tags (ESTs) available yet for *A*. *aquaticus*. Therefore, sequence information pertaining to gene coding regions is limited only to the candidate genes isolated previously [17]. To address this lack of genomic data, our primary goal is to sequence, assemble and annotate transcriptomes for *A*. *aquaticus* to identify genes associated with a range of cave-associated traits. Secondary goals include demonstrating the utility of these resources to discover genes and alleles associated with morphological and behavioral differences between cave and surface populations, which will enhance the collective database of genetic and genomic information for cave animals.

## Materials and Methods

### Transcriptome assembly

Four samples were subjected to high-throughput sequencing: the head from a surface dwelling male, the head from a cave dwelling male, the head from a hybrid male (generated from a cave male and a surface female), and around thirty pooled surface individuals from 70% of embryonic development to hatching. The pooled surface embryos and hatchlings sample was sequenced to identify genes that may be expressed during embryonic development but not in adult animals. These samples were all laboratory-reared except for the cave male which was caught and kept in captivity for several months. The founding surface population was from Planina Polje and the founding cave population from Planinska Jama (Pivka channel), both located in Slovenia. The field studies did not include any legally protected or endangered species. They were not conducted in national parks or other protected areas requiring permission under national or regional legislation. We reared animals as described previously [17]. RNA was extracted from our samples using TRIzol (Invitrogen) and the provided protocol. RNA was quantified using a Qubit Fluorometer (Invitrogen) and cDNA libraries were constructed using the SMARTer cDNA synthesis kit (Clontech). Sequencing was performed using the Roche 454 platform [19] yielding thousands of reads per sample: surface head– 184,618, cave head –75,190, hybrid head 205,502, and surface embryos/hatchlings– 171,713. This Transcriptome Shotgun Assembly project has been deposited at DDBJ/EMBL/GenBank under the accession GDKY00000000. The version described in this paper is the first version, GDKY01000000.

Comparison of assembler programs for 454 sequence data, with an emphasis for non-model systems, have suggested that SeqMan NGen (DNASTAR.v.11.0) is a program that can identify both known and novel transcripts and yields the best overall assembly [20; 21]. NGen was used to generate an integrated *de novo* transcriptome assembly from both surface and cave individuals to ensure equal representation of both morphotypes in the reference trancriptome. This assembly was generated using the recommended parameters optimized for 454 *de novo* transcriptome assembly, with the settings: matchsize = 21; match spacing = 75; minimum match percentage = 85; match score = 10; mismatch penalty = 20; gap penalty = 30; max gap = 15. We then aligned sequencing reads from surface-, cave-, hybrid- and embryo-hatching head tissue RNA pools to our reference template (the consensus sequences from the *de novo* transcriptome) for our subsequent SNP analyses.

### Transcriptome annotation

To assign identities to each of the consensus sequences from the *de novo* assembly, we utilized the online tool Blast2GO (B2G) [22]. The program uses the BLASTX algorithm through the NCBI server and the non-redundant (nr) database to collect the 20 top hits for each unknown sequence with an e-value cut-off of 1.0 x 10^−3^. Additionally, B2G retrieves all available gene ontology (GO) terms for each gene sequence. The process of GO assignment selects terms from the gene ontology database obtained through the “Mapping” step in B2G. The annotation is carried out using a stringent approach, which applies an annotation rule (AR) found on the ontology terms. This aims to identify the most specific terms with a high level of reliability. For our analyses, we employed the default settings, with the parameters: e-value hit filter: 1.0 x 10^−6^; annotation cutoff: 55; GO weight: 5; Hsp-Hit Coverage Cutoff: 0.

### Genotyping

To add genes to the existing linkage map of *A*. *aquaticus*, we first searched through the transcriptome sequences of *A*. *aquaticus* for genes that are known from the literature to be involved in either eye development or pigmentation. In addition to the *A*. *aquaticus* sequences, we also interrogated the gene sequences from other related/recent arthropod transcriptome projects including those of two other species of isopods–*Caecidotea forbesi* and *Caecidotea bicrenata* [19]. We searched through the *Caecidotea* sequences for genes involved in eye development and pigmentation because we hoped that sequences from either *Caecidotea* species would help us to design successful degenerate primers to amplify orthologous genes from *A*. *aquaticus*. After obtaining a piece of the coding sequence in *Asellus aquaticus* of each candidate gene, we isolated flanking sequences for each gene/contig using a GenomeWalker library (Clontech). Then, we identified a polymorphic marker, either a size difference or a SNP, within this sequence between cave and surface individuals and designed primers to amplify this polymorphic marker (Table 1). Next, we genotyped 8–15 individuals from our previously published cross [17] by size separation or Sanger sequencing. PCR conditions were as described [17].

**Table 1:** Primers used for genotyping the candidate genes and in the case of *Ruby*, degenerate PCR.

Primer	Sequence
*Ruby* degenerate F (Ruby degF)	ATGAARGNATHGGNATG
*Ruby* degenerate R1	TCRTCYTTYTGYTCNGGRTC
*Ruby* degenerate R2 (first)	TCNGGRCANACYTCYTCRAA
*Ruby* F	TTGCATTCATTTGGACATTTGTGGA
*Ruby* R	TGCTGTTTAGTTGATCTCTCATTGTTGTCA
*Orange* F	ATAATTTCTGTCATGTTGGTTTCCA
*Orange* R	CCCCCAGTTAAGAATCATTGATATAG
*Kynurenine formamidase* F	CCATCTCGCTGGTCTAAGAGATTTCAGC
*Kynurenine formamidase* R	TTTTTATCCGAAGCGCAAACTATGT
*Slowpoke* F	GTTCACGGCCCTCAGATCTGCTCTA
*Slowpoke* R	CCCCTTGATTGGTCTCAGAAACCTTG

### ArrayStar Analysis

To quantify the number of reads for each contig/gene in our different samples, we remapped our raw 454 sequencing reads to the reference transcriptome. With this, we identified the number of sequencing reads that mapped to each consensus sequence using the QSeq module in the program ArrayStar (DNASTAR.v.11.0). This program quantifies the raw read count as the sequencing reads that uniquely align to each reference sequence, and additionally, calculates normalized read counts with RPKM normalization methods [23]. This information allowed us to call the read depth for each SNP of each contig and was used for our allele-specific analysis.

## Results and Discussion

### *De novo* assembly of *A*. *aquaticus* samples using 454 Sequencing Technology

To increase the genetic resources available for the isopod *A*. *aquaticus*, we analyzed transcriptomes of this species. This Transcriptome Shotgun Assembly project has been deposited at DDBJ/EMBL/GenBank under the accession GDKY00000000. Similar to studies of other species that have cave and surface morphotypes, like the fish *Astyanax mexicanus* [10, 13], we produced an integrated assembly, including reads from both surface- and cave-dwelling morphs. The resulting integrated transcriptome yielded a total of 23,984 contiguous sequences, with an N50 value of 800 bp and average contig depth of 7. The contig “N50 value” is a metric used commonly to describe the overall quality of a *de novo* assembly. In short, N50 is a weighted median statistic, wherein 50% of the entire assembly has contigs equal or larger to this value.

**Fig 1:**
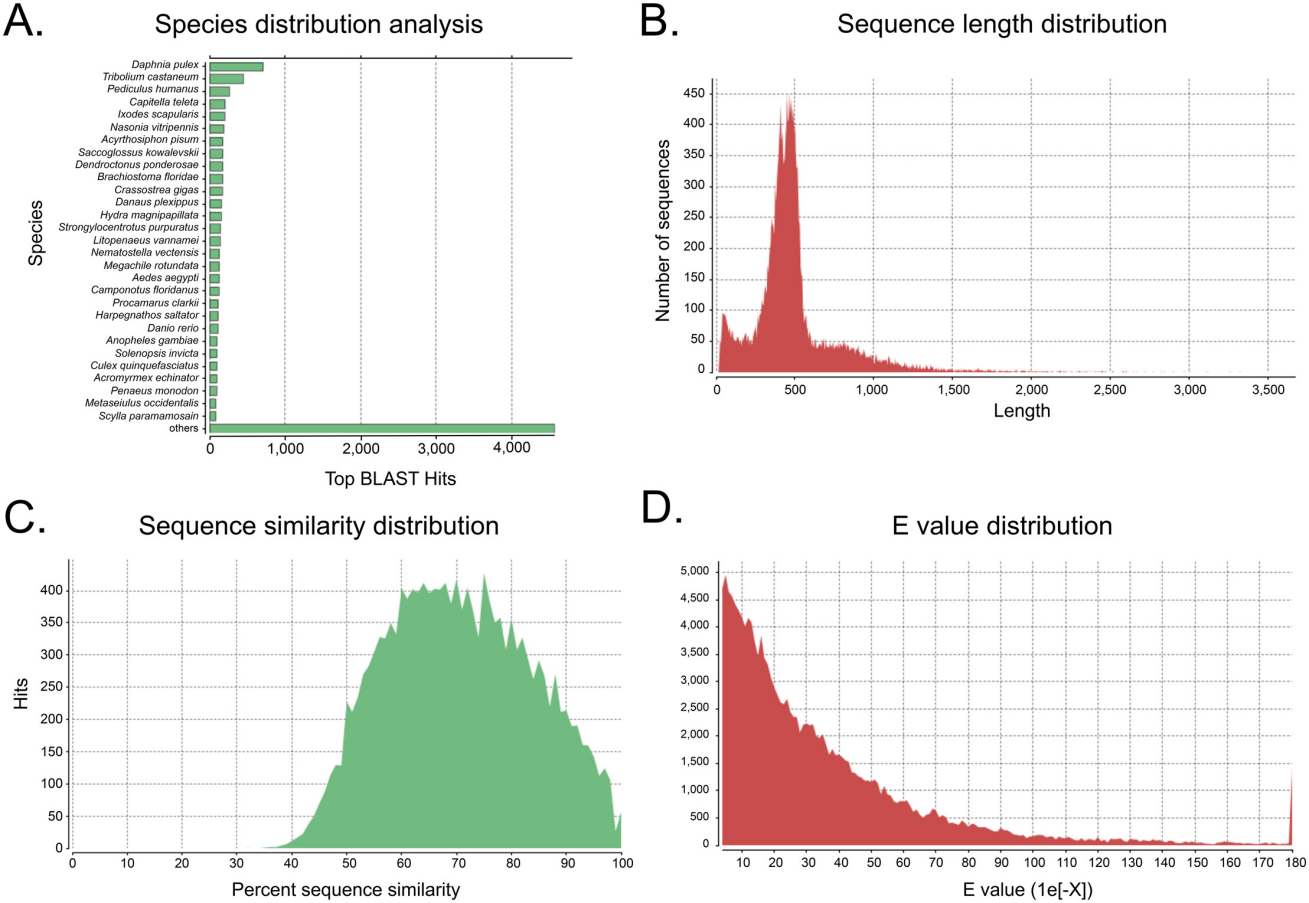
Descriptive analyses of the *A*. *aquaticus* transcriptome. A. The BLAST results reveal a wide range in matches for the top hits for each contiguous sequence, which likely is a consequence of limited genomic resources for invertebrate systems. However, the crustacean *D*. *pulex* is the species most frequently detected in our dataset. B. Our integrated trancriptome assembly yielded contigs ranging in size from 21 bp to 3490 bp, with an average length of 500 bp. C. A sequence similarity analysis further confirms the identity of genes detected in our transcriptome owing to the high level of similarity (60–80%) for each contig to their respective top hit. D. The reliability of gene identification in our transcriptome is further supported by the expect (or “e”) value distribution.

Subsequently, we performed gene annotation of our consensus sequences using the online software program Blast2GO (B2G; [22]). More than 4,000 of our BLAST hits were identified in “other” or non-model species (Fig 1A) indicating that multiple species were represented due to severe limitations of genomic resources in Crustacea [24]. However, the most common organism comprising our species distribution analysis, yielding 714 top hits, was the crustacean *Daphnia pulex*, which is the only species of crustacean with a sequenced genome [25].

The contigs from our integrated *de novo* transcriptome assembly ranged from 21 to 3490 bp (Fig 1B), with an average length of 500 bp. Further analyses demonstrated that each sequence shared 60–80% similarity with their respective top hit (Fig 1C). Again, this result is expected, considering that the most closely related available genome from the subphylum Crustacea is a member of the class Brachiopoda (*Daphnia)*, which diverged from Malacostraca (*A*. *aquaticus)* more than 380 MYa [26]. Additionally, the lower sequence similarity observed could be a result of our contigs not representing full-length gene sequences, or instead, may be an indication of substantial sequence divergence between our *Asellus* transcriptome and the available genomics resources used in our comparisons. Furthermore, lack of accessioned genomic sequences for arthropods (outside of Insecta) may account for the level of sequence divergence observed in our analyses (Fig 1D).

**Fig 2:**
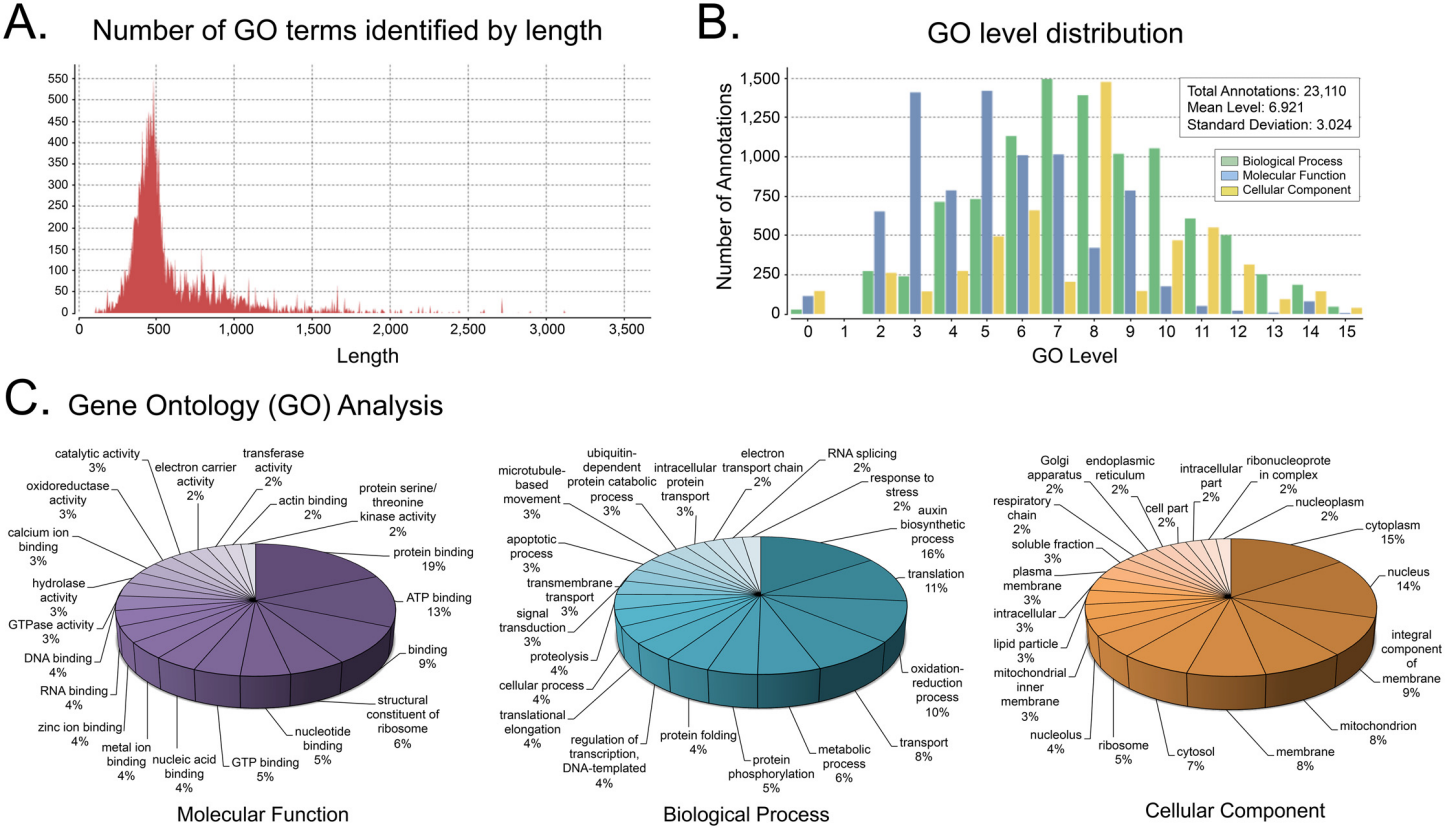
*A*. *aquaticus* gene ontology (GO) term analysis. A. Generally, gene ontology (GO) terms were assigned to sequences ranging in length from ~100 bp to ~2,500 bp. B. Over 23,000 gene ontology terms were associated with genes in the *A*. *aquaticus* transcriptome, with a median GO level of 6.921. C. The top 20 gene ontology terms represented in our transcriptome for each GO category: molecular function, biological process and cellular component.

We annotated broader functions of genes in the integrated transcriptome with Gene Ontology (GO) terms using the Blast2GO program, which collects every available GO term based on the assigned gene identity. Generally, sequences ~100–2500 bp in length were assigned gene ontology terms, with the number of potential annotations ranging from 1–500 terms for each gene (Fig 2A). Annotations were classified into three types of ontology: molecular function, biological process, and cellular component (Fig 2B). In total, over 23,000 annotations were denoted in our transcriptome, with a mean GO level of 6.921. We characterized the top 20 annotations represented in each of the three GO categories (Fig 2C). Among these annotations, the terms most evident in the molecular function, biological process, and cellular component categories were “protein binding”, “auxin biosynthetic process”, and “cytoplasm”, respectively.

### SNP discovery

One of our goals in the transcriptome analysis was to generate additional SNPs for genotypic studies. Towards this end, we screened all contigs with at least 4 reads for both cave and surface samples and retained all SNPs that were either 100% present in the cave (or surface) morphotype but not present in the other morphotype. This approach selects for informative SNPs that differentiate between the cave and surface populations. Using these parameters, we identified 1,326 SNPs (S1 Table) in 271 contigs with an average of 5 SNPs per contig.

If a SNP was fixed in the cave population and fixed in the surface population, but a different allele, we would expect the sequenced hybrid individual (which is unrelated to the surface and cave individuals) to be heterozygous for the two SNPs. Therefore, we selected only those SNPS that were not present 0 or 100% in the hybrid, and obtained 742 SNPs comprising 162 different contigs with an average of 4.5 SNPs per contig. With this, we have prioritized 742 SNPs for possible use as genetic markers. These are candidate genetic markers because the utility of these SNPs will depend on the frequency of the SNPs in the different populations. As these are wild populations, we expect that a percentage of these SNPs will not be fixed either in the cave or surface population. These SNPs could be used to add candidates to the existing linkage map to see if they coincide with loci mapped previously for traits related to pigmentation and eye morphology [17].

### Gene placement on map

We had previously placed many candidate genes on our linkage map of *A*. *aquaticus* by cloning a fragment of the gene using degenerate PCR, amplifying intronic or UTR sequence using GenomeWalker or RACE, and then designing primers to amplify polymorphic fragments (e.g., SNPs or size-length differences) between the cave and surface populations [17]. Then, we used these genetic markers to genotype a backcross composed of 194 animals using iPlex Extend and MALDI TOF mass spectrometry (Sequenom). JoinMap 4 was used to generate the linkage map [17].

Transcriptome sequencing can be used to identify polymorphic markers in genes of interest; this method identifies many candidates at once, and should identify genes that might not have enough conserved regions to design degenerate primers. To determine the location of the gene/contig on our current map, a subset of the individuals from the pedigree used to generate the linkage map can be genotyped [17] and the genotypes of the new makers and previous markers compared.

We leveraged this powerful approach to identify candidate genes for eye and pigmentation traits and place them on our linkage map. Here, we describe the addition of four new genes. We initially picked these four genes because we had prioritized the majority of them for our initial mapping study and were unsuccessful in obtaining sequence using degenerate PCR. In the future, we would like to add many more genes to the existing linkage map. We found the orthologue of *orange*, a gene in the AP-3 (adaptor protein 3) complex that has a role in cargo selective transport, in *Drosophila melangaster* [27]. *Drosophila melanogaster* mutants in the AP-3 complex harbor reduced numbers of pigment granules and decreased levels of pigment in those granules [27]. *Orange* was found in all four of the *A*. *aquaticus* transcriptomes. In addition, we obtained a sequence for the *A*. *aquaticus* orthologue of *ruby*, also an AP-3 complex member *in D*. *melanogaster* [27]. Another method used to identify candidates was Phylogenetically Informed Annotation (PIA), which annotated our transcriptome for 109 genes from the light interaction toolkit (i.e. a set of genes with roles in vision, eye morphology, and pigmentation; [19]). Nineteen of these 109 genes were found in at least one of the *A*. *aquaticus* samples: *Arr1*, *Gbeta76C*, *Gprk2*, *Gnat1*, *pinta*, *clot*, *Dhpr*, *Pcd*, *punch (Pu)*, *rosy (ry)*, *sepia (se)*, *cinnabar (cn)*, *Kfase*, *vermilion (v)*, *Alas*, *l(3)02640*, *Aldh*, *lark*, and *slowpoke* [19]. Many factors likely contribute to the lack of certain genes from the light interaction toolkit in our transcriptomes including small sample size, the sequencing technology used, and the age/ stage of individuals sequenced. Of the nineteen genes identified by transcriptome sequencing, fourteen genes had not previously been placed on the *A*. *aquaticus* linkage map [17]. We chose to add four new genes to the map, *kynurenine formamidase*, *slowpoke*, ruby, and orange. *Kynurenine formamidase* is in the ommochrome synthesis pathway [28]. There is evidence in other species of isopods, including an isopod in the same genus as *A*. *aquaticus*, *A*. *intermedius*, that ommochromes are the integumental pigments [29, 30, 31]. *Kynurenine formamidase* was found in the surface head transcriptome only. *Slowpoke* is a gene involved in the circadian clock [32]. *Slowpoke* was found in all four *A*. *aquaticus* transcriptomes.

We placed *ruby* to one of the eight *Asellus aquaticus* linkage groups, linkage group 6 near markers aa98 and aa58 which was not near any mapped loci. Similarly, we placed *orange* to linkage group 5 with identical genotypes from aa36 to aa15 10.4 cM away from the locus responsible for red versus orange/brown pigmentation (Table 2). We genotyped *slowpoke* for 16 individuals from the cross and found that the SNPs emerged with 100% agreement in one area on the map, LG7 near aa33-aa113 (Table 2). This location is near the ‘presence versus absence of eye’ locus. Similarly, we used the sequenced fragment of *kynurenine formamidase* to identify a polymorphic marker between cave and surface individuals. We found that this gene was placed on linkage group 2, near the markers aa56 and aa74, and near the locus responsible for presence versus absence of pigmentation (Table 2). We had found previously that multiple other promising candidates also map to this location on linkage group 2 such as *white*, *scarlet*, and *pale*. *White* and *scarlet* are involved in ommochrome transport [33] and, as mentioned above, ommochromes could be responsible for the integumentary pigment in *A*. *aquaticus*. Alternatively, melanins could also be the cause of integumentary pigment in *A*. *aquaticus*. *Pale* is involved in melanin synthesis and *Drosophila* mutants in *pale* are devoid in pigmentation [34]. In addition, cave plant hopper pigmentation can be rescued by the addition of one of the substrates in the melanin pathway, L-DOPA [35]. Future analyses, including sequencing of the coding region or quantifying allele-specific expression in hybrids to look for regulatory differences, can determine if any of these genes, including *kynurenine formamidase*, cause the ‘lack of pigmentation’ phenotype. In addition, placing multiple genes on the map will help anchor the linkage map if transcriptomes or genomes from a closely related species with synteny to *A*. *aquaticus* are sequenced. Furthermore, increasing the number of candidate genes on the map will help in future QTL analyses investigating additional traits and/or additional populations.

**Table 2:** Candidate gene placement on *Asellus aquaticus* linkage map.

Gene	Number of individuals genotyped	Markers in agreement	Markers with one recombinant	LG of markers in agreement	Mapped loci nearby
*Ruby*	8	aa98 and aa58		6	No
*Orange*	13	aa36, aa42, aa38, aa20, aa53, aa91, aa118, and aa15		5	10.4 cM away from red vs orange/brown pigmentation
*Kynurenine formamidase*	15		aa56, aa74	2	Within eye size and presence versus absence of pigmentation
*Slowpoke*	15	aa33, aa40, aa37, aa19, aa70, aa113		7	11.7 cM away from eye presence versus absence

Our analysis of adult transcriptomes resulted in resources that will help identify genetic differences between cave and surface populations. However, future sequencing of embryonic samples could be even more illuminating for eye and pigmentation traits depending on what genes are actually mutated and if these genes play a larger role during embryonic development than they do post-embryonically.

### Allele-specific expression in the hybrid sample

*Cis*-regulatory changes are thought to play an important role in evolutionary change [36]. Therefore, we wanted to identify genes with potential *cis*-regulatory differences between cave and surface forms. To identify *cis*-regulatory differences between cave and surface populations, we can take advantage of the fact that our hybrids contain at each locus one allele from a cave-dwelling parent and the other allele from a surface-dwelling parent. If there is significantly higher expression in that hybrid animal of one allele over another, one possibility is that the pattern is due to a *cis*-regulatory difference in the focal gene. As described in other studies, we expect regulatory changes acting in *cis* to result in allele-specific expression but regulatory changes in *trans* to not differentially affect both alleles [37, 38]

**Fig 3:**
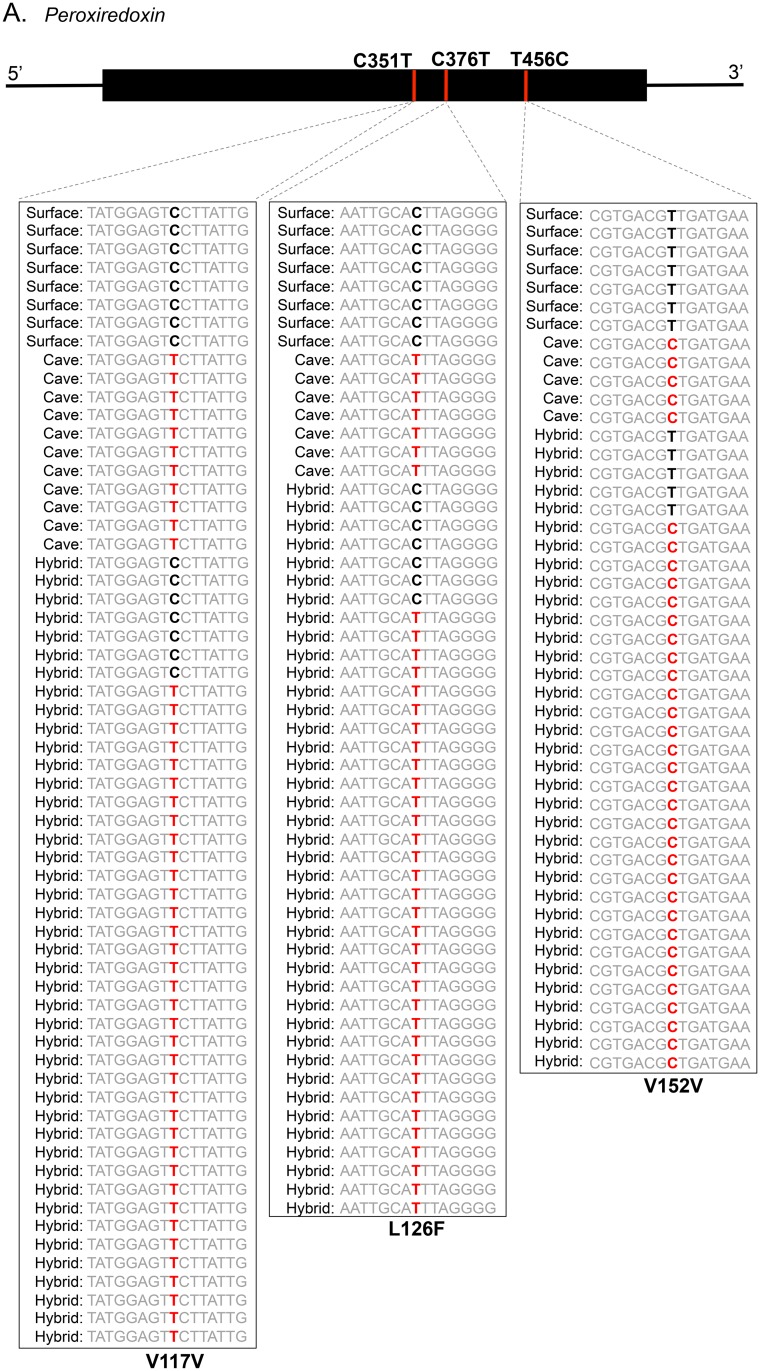
Allele-specific expression of *Peroxiredoxin-1* in a hybrid sample reveals bias for the cave-dwelling allele. A. Three SNPs were present for the contig corresponding to *Peroxiredoxin-1*. Written first is the surface nucleotide, then the position, and then the cave nucleotide. B. Shown are the number of reads for the cave, surface, and hybrid samples and the corresponding allele, either cave or surface, for each of the reads. The cave allele is shown in red and the surface allele in black. Below the reads is written whether the surface and cave alleles encode different amino acids. The first and third SNPs are sense mutations and the second results in an amino acid change.

To identify putative genes with allele-specific expression, we first screened our dataset for all contigs/genes containing a depth of at least four reads in both cave and surface samples using ArrayStar (DNASTAR.v.11.0). Then, we identified SNPs with 100% fixed differences (i.e., invariant) between the surface and cave forms. This resulted in SNPs that were likely homozygous in the surface individual and likely homozygous but different in the cave individual. For our analysis, we defined an “allele” as a contig/gene where the hybrid individual that had at least 3 SNPs that clearly demonstrated a surface or cave bias for all 3 SNPs present. In F1 hybrids, individuals have one allele from the cave and one from the surface parents. In a scenario in which both alleles are expressed equally, we would expect ~50% detection of each allele in our dataset. Therefore, we reasoned that a substantial departure from this null would be alleles demonstrating 0–30% or 70–100% surface or cave bias. Finally to ensure accurate allele-calling in the hybrid, we further filtered our dataset to only include contigs with a minimum of 6 reads per hybrid sample, and only included genes that were unique in the transcriptome (i.e., no redundancy). These parameters resulted in very few genes showing a morphotype-specific pattern, owing to the low read number per contig (see Methods). This analysis yielded five contigs, *bifunctional aminoacyl tRNA synthetase*, *tnf receptor associated*, *glutathione s transferase mu 5 like isoform*, *peroxiredoxin-1*, and *chymotrypsin like protein* showed a bias for one allele over the other using the above parameters (Fig 3; S1, S2, S3, and S4 Figs; Table 3). Future studies utilizing additional individuals and increased sequencing depth will continue to clarify the cave vs. surface allelic expression pattern of these genes in hybrid *A*. *aquaticus* offspring.

**Table 3:** Putative genes with allele specific differences.

Contig number and gene identity	Functional gene ontology (GO) category	Allele specific bias towards
Contig 2687 *peroxiredoxin 1 like*	P:cell redox homeostasis; C:cytosol; F:peroxiredoxin activity; P:oxidation-reduction process; F:peroxidase activity	Cave allele
Contig 2732 *bifunctional aminoacyl tRNA synthetase*	F: sumo binding; F: aminoacyl-tRNA ligase activity; P:tRNA aminoacylation for protein translation	Cave allele
Contig 2861 *tnf receptor associated*	F:metal ion binding; P:regulation of apoptotic process; F:zinc ion binding; P:signal transduction; F:protein binding; F:receptor activity; F:transferase activity	Cave allele
Contig 3844 *glutathione s transferase mu 5 like isoform*	F:transferase activity	Cave allele
Contig 644 *chymotrypsin like protein*	F:hydrolase activity	Surface allele

Because our transcriptome sequencing was performed in adult individuals, any putative allele-specific expression differences observed would likely explain adult phenotypes. Only a limited number of traits have been explored in adult animals, so many of the physiological, developmental, morphological and behavioral differences between the two populations remain uncharacterized. However, common cave characteristics include the presence of fewer but larger eggs, decreased metabolic rate, and increased longevity [7]. Therefore, it is not surprising that within our five genes showing allele-specific differences, we found GO term functions such as ‘oxidation-reduction processes’ and ‘hydrolase binding’. Particularly interesting are *peroxiredoxin-1 related protein* because of peroxiredoxins’ roles in longevity in flies [39] and *chymotrypsin*, a digestive protease, that could may be implicated in metabolic differences between cave and surface forms.

There are multiple reasons that one allele could have a greater number of reads than another. These reasons include methodological error, regulatory differences between populations, and parental imprinting [40]. First, we list these candidates as putative genes with allele specific differences because of the following limitations of our methodology: We used a 6X coverage because we had on average, few reads per contig, a result of using 454 technology. Because of the low coverage, there could be false positives in our list. In addition, the allele-calling was based on three individuals and additional sampling is necessary to confirm that a particular allele is fixed in each population. Furthermore, it is possible that single contigs contain multiple paralogues. Finally, the bias in expression within hybrid individuals could be an artifact of one of the alleles being preferentially sequenced, or a bias in the inclusion of alleles in the alignment. This would be more of an issue if we were mapping the reads from the hybrid to a reference transcriptome composed solely of reads from one population, for example, just the surface population [40]. However, this should not be an issue, because our *de novo* assembly is composed of both surface and cave reads. Regarding parental imprinting, additional experiments could sequence hybrids resulting from cave females and surface males, the opposite polarity of the hybrid cross implemented in this experiment. Future experiments will test these putative allele specific differences by RT-PCR or RNAseq in multiple hybrid individuals. Additional experiments will also produce a larger number of reads, allowing the use of established computational tools for examining allele specific expression [41, 42].

Furthermore, future experiments will examine differences in gene expression between samples, for example between animals from cave and surface populations. As we only sequenced one individual per population in this study, we were unable to compare gene expression levels between populations. However, sequencing multiple samples per population, in our next experiments, may allow us to investigate genes with differential expression and thereby identify both mutated genes and pathways important in the morphological and behavioral differences between the populations.

## Conclusion

We describe the first transcriptomic analysis of cave and surface dwelling populations of the isopod crustacean, *A*. *aquaticus*. Our approach led us to discover thousands of new genetic sequences for this system. We used new information on sequence variation between cave and surface populations to identify SNPs that were then used as genetic markers. We added four additional candidates to an existing linkage map, and three of these genes co-localize near associations with eye size and pigmentation variation. We further utilized this dataset to identify putative allele-specific (cave versus surface) differences within a hybrid individual, suggesting that there may be a bias for certain alleles in genes including *peroxiredoxin 1*.

This report dramatically increases the genetic resources available for *A*. *aquaticus* and provides an emerging invertebrate model of cave adaptation. This genetic sequence information will enable identification of genes and prospective genetic changes associated with differences between cave- and surface-dwelling populations. Further, a deeper understanding of the genetic changes responsible for morphological and physiological variation between cave and surface forms of *A*. *aquaticus* will ultimately enhance our knowledge of how cave animals have evolved to live in the subterranean environment.

## Supporting Information

S1 Table SNPs from transcriptomic analysis. (XLSX)

S1 Fig Putative allele-specific expression of *Glutathione s transferase mu 5 like isoform* in a hybrid. Multiple SNPs are present within the contiguous sequence predicted as *Glutathione s transferase mu 5 like isoform*. The surface allele reads are in black and the cave allele reads in red.
(TIF)

S2 Fig Putative allele-specific expression in *Bifunctional aminoacyl tRNA synthetase*. The contig corresponding to *Bifunctional aminoacyl tRNA synthetase* demonstrates three SNPs.
(TIF)

S3 Fig Putative allele-specific expression for *Tnf receptor associated*. Shown are numerous SNPs detected in the contig for *Tnf receptor associated*.
(TIF)

S4 Fig Putative allele-specific expression for *Chymotrypsin like protein*. Five SNPs are shown in *Chymotrypsin like protein*.
(TIF)
