# Supplementary figures and images for: A Transcriptomic Analysis of Cave, Surface, and Hybrid Isopod Crustaceans of the Species Asellus aquaticus
Source: PLoS One. 2015 Oct 13;10(10):e0140484. doi: 10.1371/journal.pone.0140484 (PMC4604090; doi:10.1371/journal.pone.0140484)

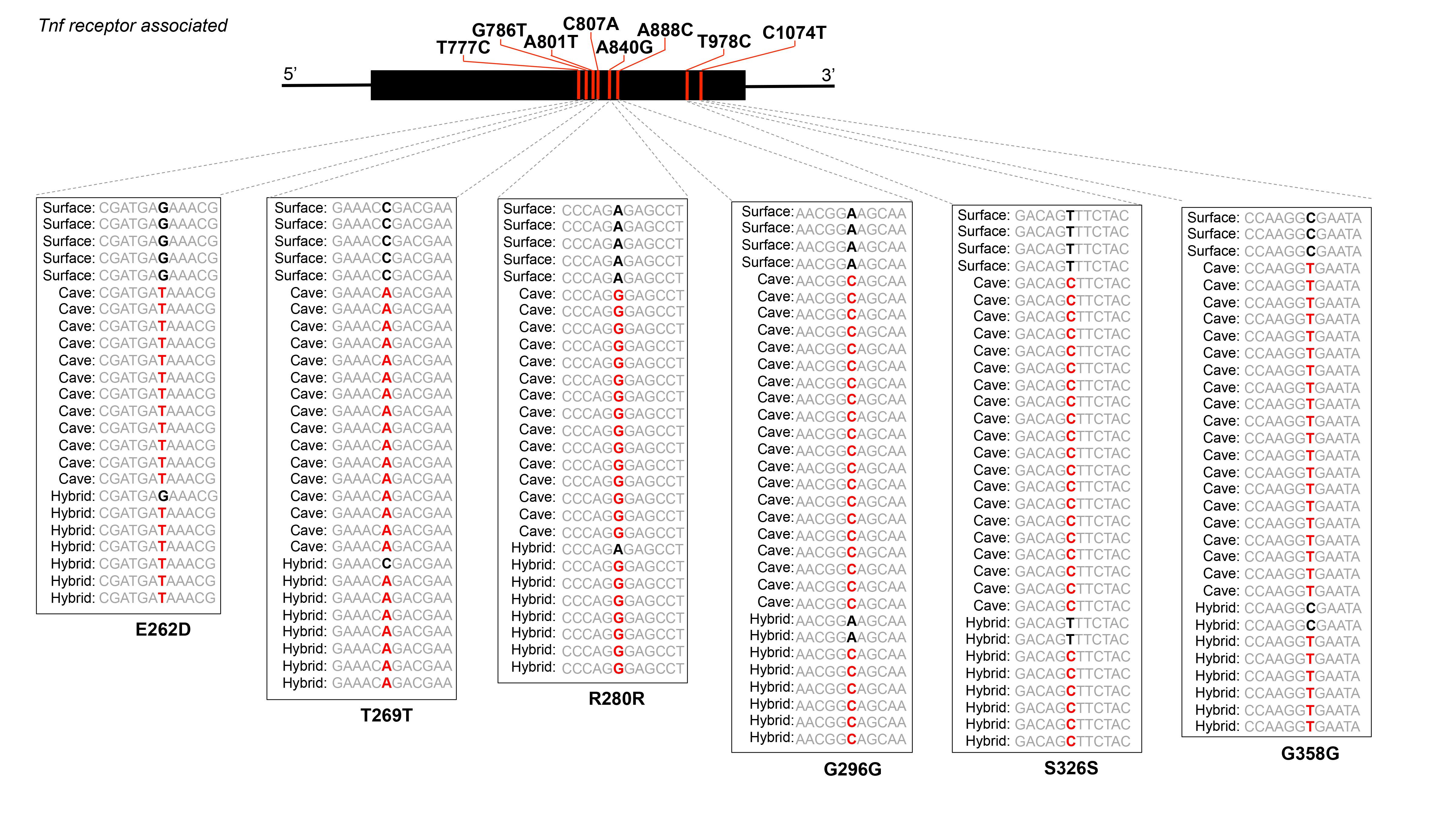

Supplement: S1 Fig — Multiple SNPs are present within the contiguous sequence predicted as Glutathione s transferase mu 5 like isoform. The surface allele reads are in black and the cave allele reads in red. (TIF) [file pone.0140484.s002.tif]

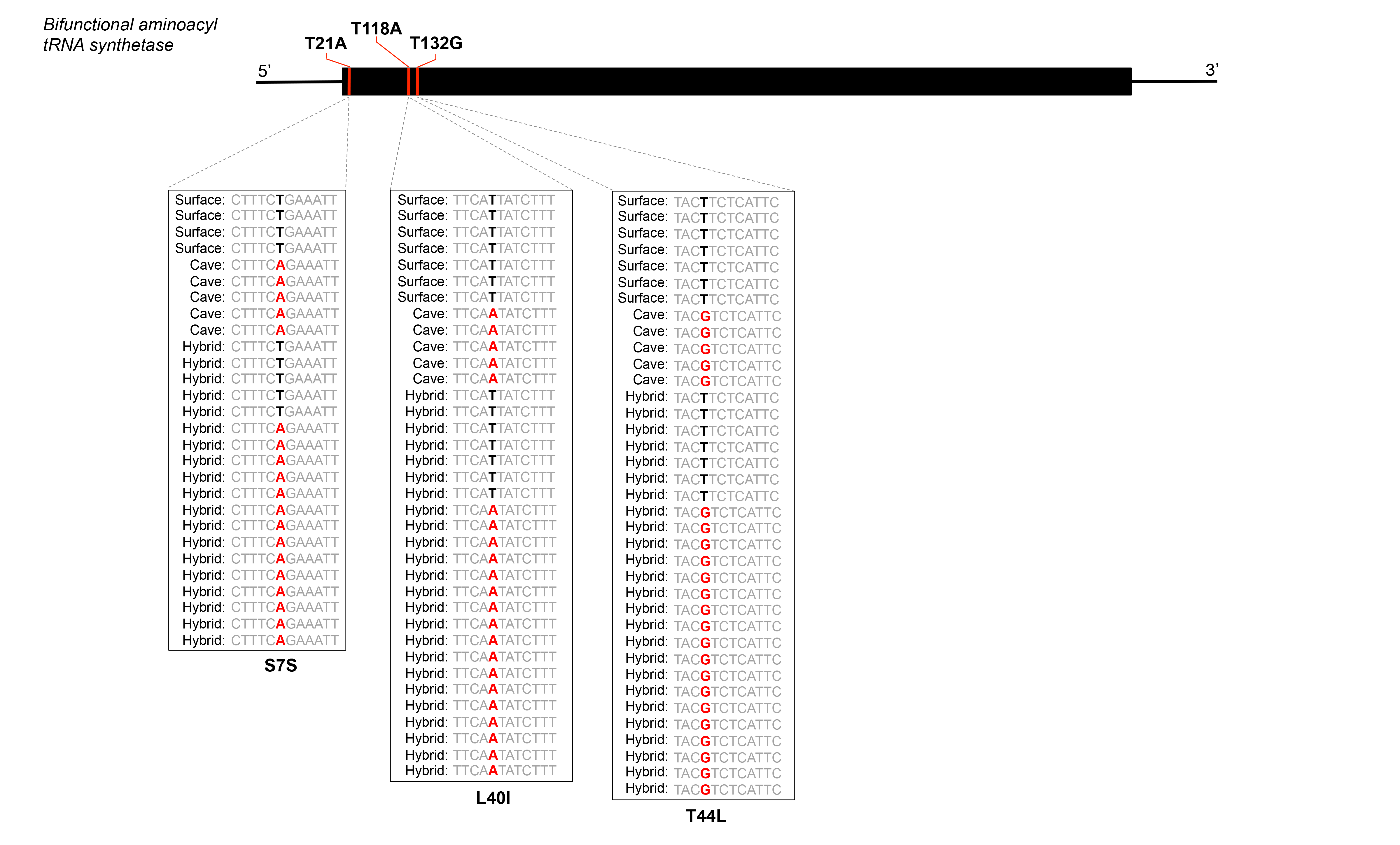

Supplement: S2 Fig — The contig corresponding to Bifunctional aminoacyl tRNA synthetase demonstrates three SNPs. (TIF) [file pone.0140484.s003.tif]

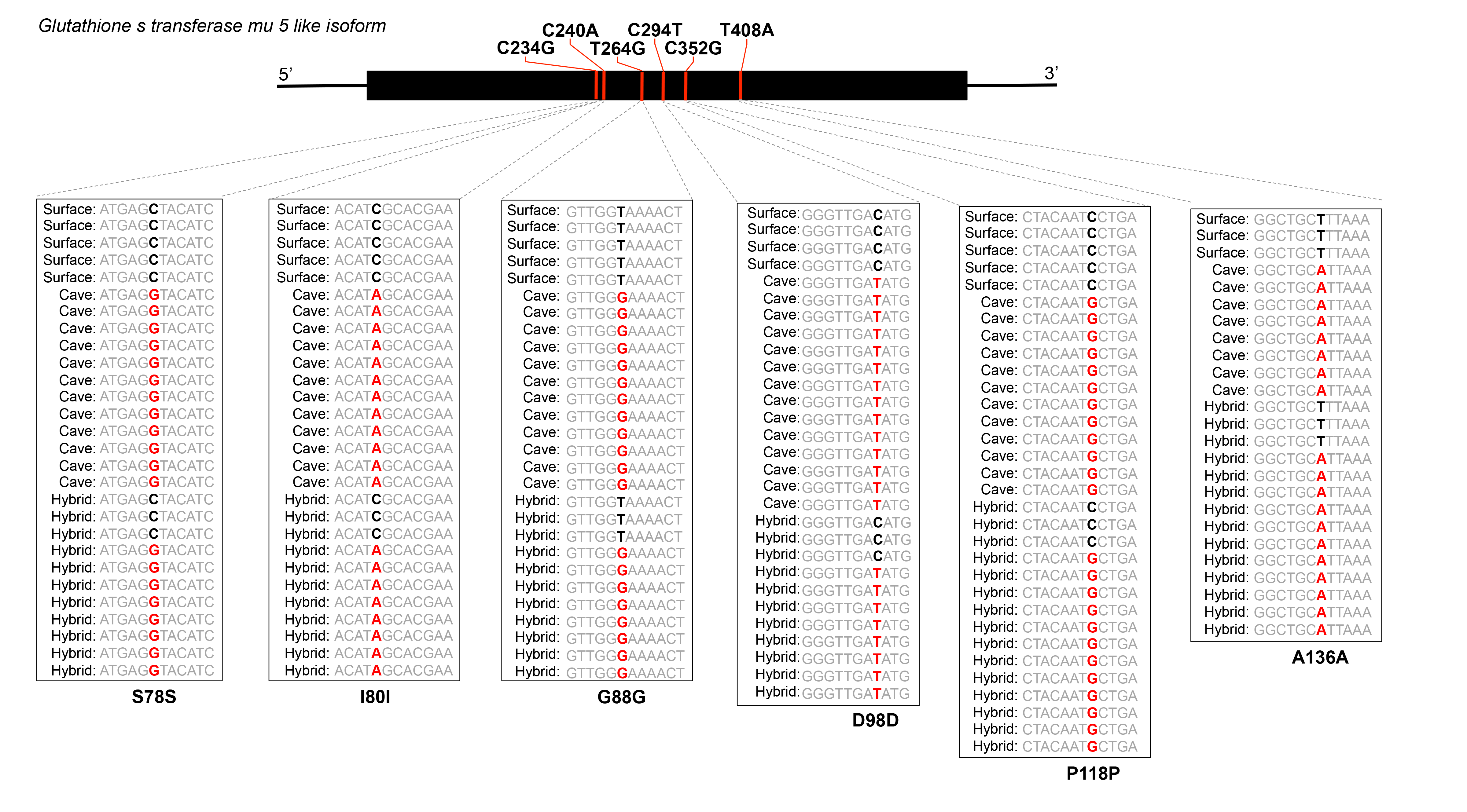

Supplement: S3 Fig — Shown are numerous SNPs detected in the contig for Tnf receptor associated. (TIF) [file pone.0140484.s004.tif]

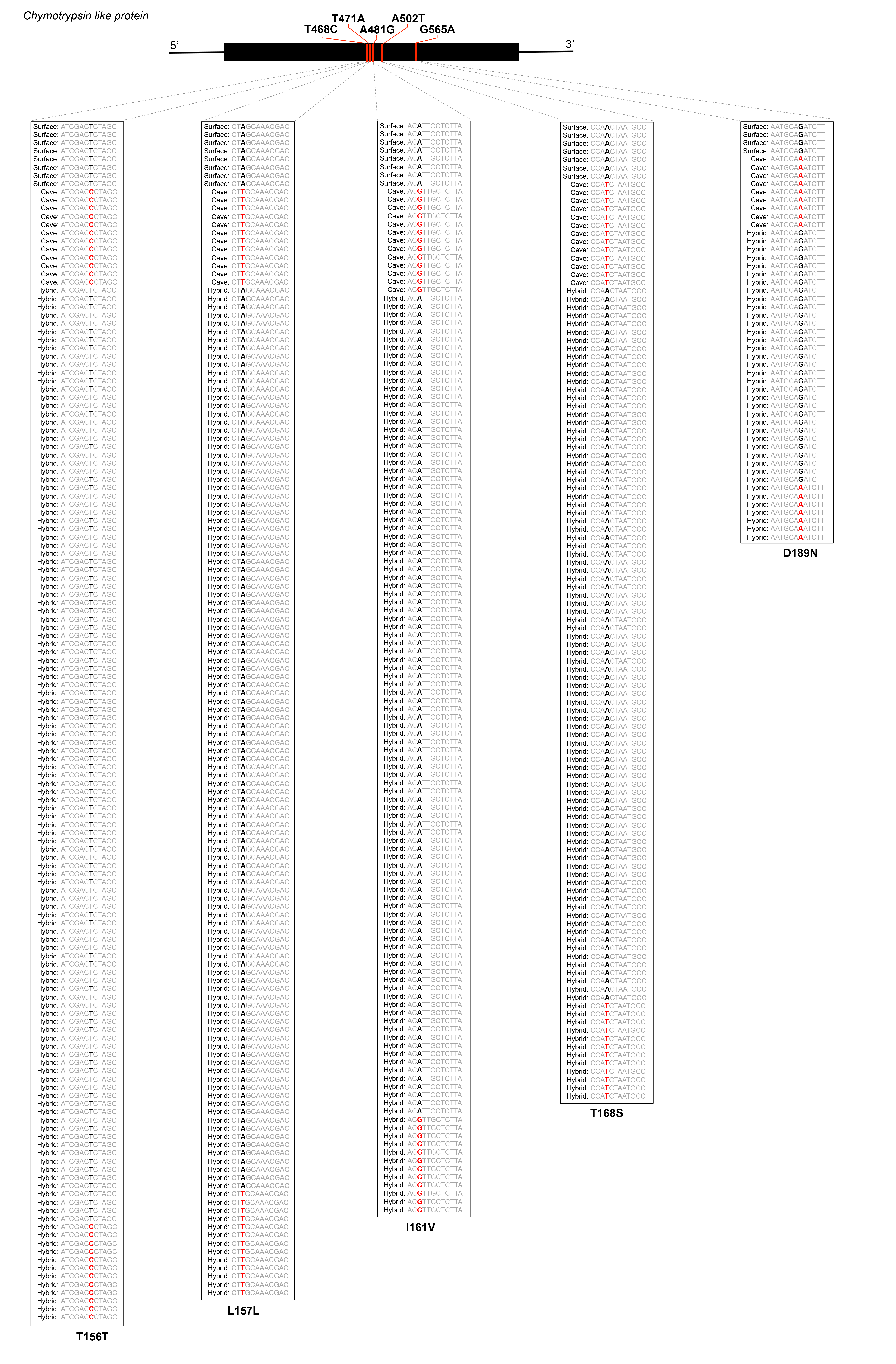

Supplement: S4 Fig — Five SNPs are shown in Chymotrypsin like protein. (TIF) [file pone.0140484.s005.tif]
